# Praziquantel-related visual disorders among recipients in mass drug administration campaigns in schistosomiasis endemic settings: Systematic review and meta-analysis protocol

**DOI:** 10.1371/journal.pone.0300384

**Published:** 2024-05-17

**Authors:** Anthony Danso-Appiah, David Owiredu, Kwadwo Owusu Akuffo

**Affiliations:** 1 Centre for Evidence Synthesis and Policy, University of Ghana, Legon, Accra, Ghana; 2 Department of Epidemiology and Disease Control, School of Public Health, University of Ghana, Legon, Accra, Ghana; 3 Department of Optometry and Visual Science, College of Science, Kwame Nkrumah University of Science and Technology, Kumasi, Ghana; Universite Felix Houphouet-Boigny, SWITZERLAND

## Abstract

**Background:**

Hundreds of millions of doses of Praziquantel (PZQ) have been administered to persons with and without schistosomiasis living in schistosomiasis endemic settings, through the mass drug administration (MDA) strategy which started in the early 2000s. A recent publication suggested high risk of PZQ-related visual disorders, raising public health concerns. We aim to systematically synthesize evidence on the magnitude of PZQ-related visual disorders.

**Methods:**

We will search PubMed, Google Scholar, CINAHL, SCOPUS, CENTRAL and LILACS from 1977 (when the first human clinical trials on PZQ started) to 31^st^ May 2024, with no language restrictions. The key search terms will include “Praziquantel”, “PZQ”, “visual disorder”, “adverse events”, “side effects”, “blurry vision” and “visual impairment” together with alternative terms and synonyms. All the countries endemic for schistosomiasis will be included as search terms. We will also search HINARI, Africa Journals Online, Thesis Databases and Preprint Repositories. Where necessary, we will contact expert researchers working in the field of schistosomiasis, UNICEF/UNDP/World Bank/WHO Special Programme for Research and Training in Tropical Diseases (TDR), pharmaceutical industries, country-specific Food and Drug Authorities (FDAs) and the European Medicines Agency databases. We will search Conference Proceedings and reference lists of relevant studies for additional studies. At least two authors will independently select studies, extract data and assess risk of bias in the included studies. Any disagreements or discrepancies will be resolved through discussion between the reviewers. Heterogeneity will be explored graphically, and statistically using the I^2^-statistic. We will conduct random-effects meta-analysis when heterogeneity is appreciable, and express dichotomous outcomes (visual adverse events including excessive lacrimation, blurry vision and visual impairments) as risk ratio (RR) or Odds Ratio (OR) with their 95% confidence interval (CI). We will perform subgroup analysis to assess the impact of heterogeneity, and sensitivity analyses to test the robustness of the effect estimates. The overall level of evidence will be assessed using GRADE.

**Expected outcomes:**

The present review expects to identify and categorize visual disorders occurring after administration of PZQ, alone or in combination with other drugs. By synthesizing the data from multiple studies, the review aims to present a quantitative assessment of the risk or odds of experiencing a visual disorder in different populations after ingesting PZQ. The review will also generate insights into whether PZQ in combination with other drugs are associated with increased odds of visual disorders and whether the occurrence of visual disorders correlates with dosage or treatment duration. Policymakers, public health experts and stakeholders could rely on the review findings to deliver context-sensitive preventive chemotherapy programs by adjusting drug combinations or dosing schedules to reduce risk of visual adverse effects in populations treated with PZQ. The review aims to identify gaps in the current evidence regarding visual disorders following PZQ administration in schistosomiasis endemic settings which can serve as the basis for future research on important but unanswered questions.

**Dissemination and protocol registration:**

The findings of this study will be disseminated through stakeholder forums, conferences, and peer-review publications. The review protocol has been registered in the International Prospective Register for Systematic Reviews (PROSPERO)- CRD42023417963.

## Background

Schistosomiasis affects millions of people worldwide [1]. The global response to eliminating the disease led to the rollout of preventive chemotherapy (PC), the control strategy recommended by WHO and started in the early 2000s, is applied in many endemic countries [1–9]. Several global control intervention initiatives have evolved since then into large-scale population-based mass drug administration helminthic preventive chemotherapy campaigns with safe, effective, cheap and easy-to-administer drugs [2–4, 6, 10, 11]. Central to these programs is the control of established infection and prevention of new cases or transmission of the infection by disrupting its life cycle [5, 9, 11, 12].

Praziquantel (PZQ), a broad-spectrum drug against schistosome infections in humans and other animals, has over the years been used as the treatment of choice for schistosomiasis [11, 13–15]. Following the synthesis of evidence through a Bayesian systematic review and meta-analysis that addressed speculations of emerging resistance against the drug [16], the development and use of PZQ has been widely accepted as the viable preventive treatment for schistosomiasis till date [15]. Hundreds of millions of doses of PZQ have been administered to people with and without schistosomiasis living in endemic settings earmarked for MDA programmes [1, 11, 17]. In 2021, it was estimated that over 251 million people required PZQ treatment worldwide [1].

PZQ combines a unique broad-spectrum with easy and safe application at a low price [2, 18, 19]. Once ingested, the drug penetrates the tegument of the schistosome and swiftly moves through the tissues of the parasite leading to destruction of the parasite muscles [20]. One shortfall, despite its high efficacy, is its ineffectiveness against immature worms [21, 22]. The apparent benefits of PZQ chemotherapy would be the reduction in morbidity of affected persons and interruption of transmission of the infection.

Though a number of side effects such as dizziness, diarrhea and abdominal pain have been reported after PZQ administration, these have often been mild and mostly self-limiting, rarely requiring further treatment or hospitalization [23–26]. However, a recent report has suggested that PZQ may be associated with visual disorders [27]. In their report, MDA with PZQ (Biltricide, 600 mg tablets) in Eritrea led to 61 cases of visual disorders within the first 24 hours among 2,579 individual case safety reports of various adverse drug reactions although these were mostly transient. The study reported a causal relationship between PZQ use and visual disorders after what they called critical assessment of the available data [27]. In a formal comment on their publication, Montresor et al. (2020) [28] stressed that visual adverse effects had not been reported in the past following PZQ administration, probably, as a result of inadequate surveillance and highlighted four key limitations in the article by Debesai & Russom (2020) as follows: 1) the nature of assessment of the visual disorders; 2) the manner of signs and/or symptoms of the disorders; 3) the extent of resolution of the signs and/or symptoms and how they were evaluated; and 4) key characteristics of the individuals that were affected such as body weight and height. Montresor and colleagues also posited that some of the other adverse events reported by Debesai & Russom (2020) could be the results of an increase in intracranial pressure, which implicates cerebral involvement after receiving PZQ treatment. However, the commenters affirmed that the evidence is not conclusive as, still, there are uncertainties around this topic.

As a neglected tropical disease (NTD) that affects millions of people, particularly in low-income and developing countries [29], schistosomiasis control remains a key objective to achieving Sustainable Development Goal Three (SDG 3)- good health and well-being, target 3.3 which stipulates ending epidemics of HIV/AIDS, tuberculosis, malaria and neglected tropical diseases [30]. Continued assessment of the safety of PZQ constitutes an important aspect of maintaining its availability (as the best chemotherapy) and public acceptability, which is invariably crucial to achieving the ambitious elimination targets. Also, schistosomiasis has been shown to significantly impact children’s education due to its effects on health and cognitive development [31–36]. All efforts geared towards addressing this disease thus contribute to creating a better learning environment for children and ensuring universal access to quality education- SDG 4, target 4a.

The safety of PZQ has been extensively studied. In fact, there are several systematic reviews that have sought to synthesize evidence on the efficacy and tolerability of PZQ administered alone or in combination with other medicines (such as Albendazole) in school-aged children (SAC), preschool-aged children (PSAC), and pregnant women [15, 24, 26, 37–41]. Overall, available evidence from these reviews suggests that PZQ is safe and effective, with a high efficacy maintained for over four decades [21]. However, recent concerns over the occurrence of visual disorders following ingestion of the drug [27] warrants a comprehensive curation and collation of the available evidence on this important adverse effect of PZQ. To the best of our knowledge, there is no existing systematic review that has pursued the outcomes sought after by the current review.

The current review seeks to synthesize available evidence on visual disorders following PZQ administration alone or co-administered with other drugs such as Mebendazole, Pyrantel pamoate or Levamisole (for soil-transmitted helminthiasis), or Albendazole plus either Ivermectin or Diethylcarbamazine (DEC) for lymphatic filariasis, or Artemisinin-Combination Therapy (ACTs) for malaria or Azithromycin (Zithromax) for trachoma during preventive chemotherapy in endemic settings.

## Materials and methods

This systematic review protocol has been prepared using the Preferred Reporting Items for Systematic Reviews and Meta-Analyses for protocol (PRISMA-P) guidelines [42] and the full review will be conducted in line with the PRISMA guidelines (S1 Table) [43]. Comprehensive search methods, best practices and robust methods will be employed in the conduct of the systematic review (selection of studies, extraction of data and assessment of risk of bias in the included studies. The review will adopt the PICOS framework (P-Patient, I-intervention, C-control, O-Outcomes, S-Study) to describe the population, intervention, comparators, outcome of intervention and study types of the primary studies. Adverse outcomes, which are the primary focus of the review will be quantified, where possible, and classified based on severity (as mild, moderate or severe). The incidence of serious adverse events will be defined as any fatal or life-threatening event or event requiring hospitalization, or resulting in disability [44, 45]. The review has been registered in PROSPERO—CRD42023417963.

### Criteria for considering studies for inclusion in this review

#### Types of studies

Any study, published or unpublished, that assessed visual disorders associated with PZQ, will be eligible for inclusion. Reviews and commentaries or opinions will not be eligible for inclusion. For relevant reviews, we will go through the full text and reference list to identify potentially eligible studies missed by our searches. In the case where the results of a multi-center or multi-country study have been pooled, we will attempt to separate the data by site or country. However, where the results of a multi-centre or multi-country study have been lumped together and there is no way of extracting data for the independent centres or countries, or obtaining the raw data, such a study will be excluded from the meta-analysis.

#### Types of participants

Individuals (school age children, adults including pregnant and lactating women) living in endemic areas, whether infected or not infected, who received PZQ at any dose through active case detection, mass drug administration via preventive chemotherapy or received PZQ in health facilities, will be eligible for inclusion. Studies involving participants who cannot talk for themselves will be excluded.

#### Intervention

PZQ given alone at any dosing schedule or co-administered with interventions for mass drug administration for other communicable diseases co-endemic with schistosomiasis such as albendazole, mebendazole, pyrantel pamoate or levamisole (for soil-transmitted helminthiasis), or albendazole plus either ivermectin or diethylcarbamazine (DEC) (for lymphatic filariasis), Artemisinin-Combination Therapy (ACT) (for malaria), or Azithromycin (Zithromax) (for trachoma) will be eligible for inclusion.

#### Control

No treatment, placebo, or other drug regimen co-administered with PZQ will be considered as the comparator.

#### Outcomes

Primary adverse events*.

Visual disorders (blurry vision, visual impairments, lacrimation etc.).

Secondary adverse events*.

Any other visual adverse events assessed and reported in the primary study*.

* Profile of adverse events including those that are a consequence of the drug’s action after killing the parasites among infected and uninfected persons, frequency of adverse events, resolution of adverse events, and incidence of serious adverse events (SAE) will be explored. We will attempt to capture all visual disorders (short- and long- term) after PZQ administration (by a questionnaire or when spontaneously reported by the persons themselves). However, given that recall bias could occur particularly for mild events and when the period between the MDA and questionnaire survey is long (for example several years), we will categorize and analyse events according to when information was collected or spontaneously reported after receiving PZQ.

### Search methods

We will search the following databases using the search strategy described in Table 1: PubMed, SCOPUS, Google Scholar, CINAHL and LILACS from 1977 (when the first human clinical trials on PZQ started) to 31^st^ May 2024, with no language restrictions. The key search terms include “Praziquantel”, “PZQ”, “adverse events”, “side effects”, “visual disorder”, “blurry vision”, “visual impairments” and lacrimation and these will be nested with their applicable alternative terms and synonyms using the Boolean logic to provide a comprehensive search strategy. All the countries endemic for schistosomiasis will be included as search terms. The Cochrane Infectious Diseases Group Specialized Register, Cochrane Central Register of Controlled Trials (CENTRAL), published in the Cochrane Library 2024, mRCT, HINARI and African Journals Online as well as Thesis Databases, Preprint Repositories and Conference Proceedings will be searched for more studies. The bibliographies of all relevant articles including systematic reviews will be searched for additional studies. Where necessary, we will contact individual researchers working on antischistosomal drugs, pharmaceutical industries and experts from the UNICEF/UNDP/World Bank/WHO Special Programme for Research and Training in Tropical Diseases (TDR) for unpublished data and ongoing trials.

**Table 1:** Search strategy for PubMed (to be tailored to other databases).

Search	Query
#1	Search: (((((((((((((((((((((((((Schistosom*[Title/Abstract]) OR ("Schistosome infection*"[Title/Abstract])) OR (Bilharzia*[Title/Abstract])) OR ("bloody urine"[Title/Abstract])) OR (Haematuria[Title/Abstract])) OR (Hematuria[Title/Abstract])) OR (microhematuria[Title/Abstract])) OR (microhaematuria[Title/Abstract]) OR ("snail fever"[Title/Abstract])) OR ("Katayama fever"[Title/Abstract])) OR ("trematode infection*"[Title/Abstract])) OR ("Schistosoma haematobium"[Title/Abstract])) OR ("S. haematobium"[Title/Abstract])) OR ("Schistosoma hematobium"[Title/Abstract])) OR ("S. hematobium"[Title/Abstract]) OR ("Schistosoma mansoni"[Title/Abstract])) OR ("S. mansoni"[Title/Abstract]) OR ("Schistosoma japonicum"[Title/Abstract])) OR ("S. japonicum"[Title/Abstract])) OR ("Schistosoma intercalatum"[Title/Abstract])) OR ("S. intercalatum"[Title/Abstract])) OR ("Schistosoma mekongi"[Title/Abstract])) OR ("S. mekongi"[Title/Abstract])) OR ("Schistosome species"[Title/Abstract])) OR ("blood fluke*"[Title/Abstract])))))
#2	Search: (schistosomiasis[MeSH Terms]) OR (schistosomiases[MeSH Terms])
#3	Search: (#1) OR (#2)
#4	Search: Praziquantel[Title/Abstract] OR Biltricide[Title/Abstract] OR Pharmamed[Title/Abstract] OR Distocide[Title/Abstract]
#5	Search: "mass drug administration"[Title/Abstract] OR "mass treatment"[Title/Abstract] OR "preventive chemotherapy"[Title/Abstract] OR chemotherapy[Title/Abstract] OR treatment[Title/Abstract] OR "Blanket treatment"[Title/Abstract] OR MDA[Title/Abstract]
#6	Search: ((Algeria[Title/Abstract] OR Angola[Title/Abstract] OR "Antigua and Barbuda"[Title/Abstract] OR Benin[Title/Abstract] OR Botswana[Title/Abstract] OR Brazil[Title/Abstract] OR "Burkina Faso"[Title/Abstract] OR Burundi[Title/Abstract] OR Cambodia[Title/Abstract] OR Cameroon[Title/Abstract] OR "Central African Republic"[Title/Abstract] OR Chad[Title/Abstract] OR China[Title/Abstract] OR Congo[Title/Abstract] OR "Côte d’Ivoire"[Title/Abstract] OR "Democratic Republic of the Congo"[Title/Abstract] OR Djibouti[Title/Abstract] OR "Dominican Republic"[Title/Abstract] OR Egypt[Title/Abstract] OR "Equatorial Guinea"[Title/Abstract] OR Eritrea[Title/Abstract] OR Eswatini[Title/Abstract] OR Ethiopia[Title/Abstract] OR Gabon[Title/Abstract] OR Gambia[Title/Abstract] OR Ghana[Title/Abstract] OR Guadeloupe[Title/Abstract] OR Guinea[Title/Abstract] OR "Guinea-Bissau"[Title/Abstract] OR India[Title/Abstract] OR Indonesia[Title/Abstract] OR Iran[Title/Abstract] OR Iraq[Title/Abstract] OR Japan[Title/Abstract] OR Jordan[Title/Abstract] OR Kenya[Title/Abstract] OR "Lao People’s Democratic Republic"[Title/Abstract] OR Lebanon[Title/Abstract] OR Liberia[Title/Abstract] OR Libya[Title/Abstract] OR Madagascar[Title/Abstract] OR Malawi[Title/Abstract] OR Malaysia[Title/Abstract] OR Mali[Title/Abstract] OR Martinique[Title/Abstract] OR Mauritania[Title/Abstract] OR Mauritius[Title/Abstract] OR Montserrat[Title/Abstract] OR Morocco[Title/Abstract] OR Mozambique[Title/Abstract] OR Namibia[Title/Abstract] OR Niger[Title/Abstract] OR Nigeria[Title/Abstract] OR Oman[Title/Abstract] OR Philippines[Title/Abstract] OR "Puerto Rico"[Title/Abstract] OR Rwanda[Title/Abstract] OR "Saint Lucia"[Title/Abstract] OR "Sao Tome and Principe"[Title/Abstract] OR "Saudi Arabia"[Title/Abstract] OR Senegal[Title/Abstract] OR "Sierra Leone"[Title/Abstract] OR Somalia[Title/Abstract] OR "South Africa"[Title/Abstract] OR "South Sudan"[Title/Abstract] OR Sudan[Title/Abstract] OR Suriname[Title/Abstract] OR "Swaziland"[Title/Abstract] OR "Syrian Arab Republic"[Title/Abstract] OR Thailand[Title/Abstract] OR Togo[Title/Abstract] OR Tunisia[Title/Abstract] OR Türkiye[Title/Abstract] OR Turkey[Title/Abstract] OR Uganda[Title/Abstract] OR Tanzania[Title/Abstract] OR Venezuela[Title/Abstract] OR Yemen[Title/Abstract] OR Zambia[Title/Abstract] OR Zimbabwe[Title/Abstract]))) OR ()
#7	Search: (#3) AND (#4)
#8	Search: (#5) AND (#7)
#9	Search: (#6) AND (#8)

#### Managing the search output and selecting studies

**Fig 1:**
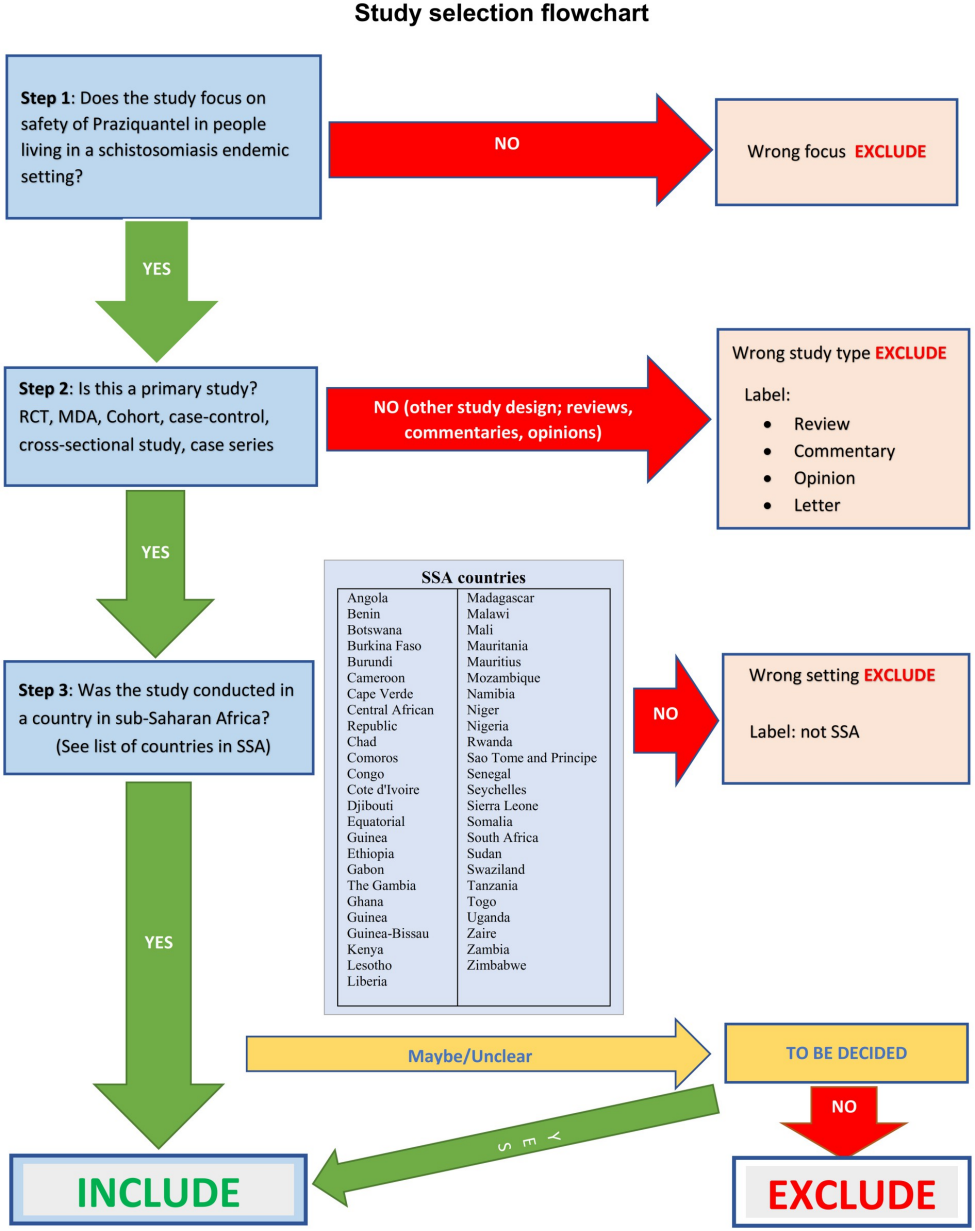
Study selection flow chart.

We will upload all references identified through our searches (electronic database and additional searches) into Mendeley where duplicates will be removed. The remaining references will be exported to Rayyan [46] where screening and selection of studies will be carried out by at least two of the reviewers independently using pretested study selection flow chart developed from the inclusion/exclusion criteria (Fig 1). First, titles and abstracts will be screened and those articles that meet the inclusion criteria will be listed and full text will be obtained for further assessment for inclusion/exclusion. The PRISMA Flow Diagram [43] (S1 Fig) will be used to document the flow of studies and reasons for exclusion. Any disagreements between screening authors will be resolved through discussion.

### Data extraction and management

At least two reviewers will extract data using a pretested data extraction form designed in Microsoft Excel [47]. Data to be extracted include study characteristics such as the study ID, country and year in which the study was conducted, the study design and the methods; information on PZQ-related visual disorders or adverse events such as blurry vision, visual impairments and lacrimation and other clinical presentation; biochemical or pathological untoward outcomes; and severity (as mild, moderate, severe) and serious adverse events (any fatal or life-threatening event, or event requiring hospitalization, or resulting in a disability); and epidemiological data–e.g. endemicity status, region where the study was conducted, participants’ prior treatment status, target population, sex, age and number of participants. Where necessary, authors of primary studies will be contacted for clarification or additional data. If two or more communities were involved in a study, data will be extracted for each community–with a link to the parent study. However, where it is not possible to separate the data, the study will be presented as one and the communities involved will be shown. We will attempt to collect complete data on all pertinent variables including PZQ dosing schedules, drug combinations, duration of treatment and visual adverse events and whether the events resolved spontaneously or required further intervention. In cases of missing data, authors of the primary studies will be contacted for the possibility of providing the needed information. Missing data will not be inputted through any computational means. Any discrepancies will be resolved through discussion between the reviewers.

### Assessment of risk of bias in the included studies

For randomized controlled trial (RCTs), the Cochrane Risk of Bias Tool (RoB 2) [48] will be used. All the five main domains, thus: 1) risk of bias arising from the randomization process; 2) risk of bias due to deviations from the intended interventions; 3) risk of bias arising from missing outcome data; 4) risk of bias in measurement of the outcome; and 5) risk of bias in the selection of the reported result; will be considered in assessing the methodological quality of included RCTs. For each domain, a judgment of ‘low risk of bias’, ‘high risk of bias’ or ‘some concerns of bias” will be made. ROBINS-I (Risk of Bias in Non-randomised Studies—of Interventions) [49], will be used to assess risk of bias in estimates of the comparative harm or benefit of interventions from studies that did not use randomisation to allocate units (individuals or clusters of individuals) to comparison groups. Seven domains through which bias might be introduced into a Non-randomised Studies of Interventions (NRSI) will be considered. The first two domains, covering confounding and selection of participants into the study, address issues before the start of the interventions that were compared (“baseline”). The third domain addressed classification of the interventions themselves. The other four domains addressed issues after the start of interventions: biases due to deviations from intended interventions, missing data, measurement of outcomes, and selection of the reported result [49]. The Risk of Bias Tool for Prevalence Studies [50] will be used to assess the methodological quality for observational studies. Where information in the study is unclear, we will contact the authors for clarification. Any inconsistencies will be resolved through discussion between the reviewers.

### Data synthesis, heterogeneity assessment and sensitivity analysis

The absolute rates of visual adverse events will be calculated from all included studies and relative risks from comparative trials. Data will be analysed and presented as risk ratio (RR) or Odds Ratio (OR) (for dichotomous outcomes) or mean difference (MD) with standard deviation (SD) (for continuous outcomes), each effect measure expressed with their 95% confidence intervals (CIs). Meta-analyses will be performed using Cochrane’s Review Manager v5.4. We will assess heterogeneity by inspecting the forest plots for overlapping CIs and outliers; using the Chi^2^ test with a P value < 0.1 to indicate statistically significant heterogeneity [51, 52]. Although a P-value below 0.05 is generally considered to indicate statistical significance [53], we will use a more sensitive threshold–i.e. a P-value below 0.10, to indicate statistically significant heterogeneity. The *I*^*2*^ statistic which describes the percentage of variation across studies that is due to heterogeneity rather than chance [54] will be inspected and categorized into 25%, 50% and 75% representing low, medium and high heterogeneity, respectively. When significant heterogeneity is detected, we will use a random-effects model; otherwise, a fixed-effect model will be used. We will present in a table, visual adverse events and all other data that cannot be meta-analysed. We will conduct subgroup analyses to evaluate potential causes of heterogeneity by stratifying the analyses by age, background endemicity, and type of schistosome species. The confidence intervals of the different resultant estimates will then be compared and interpreted accordingly [55]. We plan to carry out sensitivity analysis to assess the impact of outlier studies included in the meta-analysis (for example, studies with large sample sizes) on the robustness of the pooled estimates. This will help gauge the strength of the pooled estimates.

### Strengths and limitations of this review

- This systematic review will not rely on clinical trial data only but also observational studies including case series, case reports, country-specific Food and Drugs Authorities reports and the European Medicines Agency records accruing early and post-marketing clinical trials data.
- Pooled analysis of a number of individual studies and information from national and international repositories may enable the identification of missed serious or rare but clinically important adverse events following PZQ ingestion.
- The study will use a comprehensive search strategy that combines a number of relevant electronic databases and non-database sources to attempt to retrieve all potentially relevant studies meeting our pre-specified eligibility criteria.
- A possible limitation of this systematic review could be the lack of spontaneous reporting by persons who suffer an adverse events or lack of quality of the primary studies to be included in the review.

### Ethics and dissemination

Ethical approval was inapplicable as this study was a secondary research drawing from existing studies which received ethical clearance for their respective studies. The results of this study will be made widely available and accessible to programme managers, practitioners, patients and policy makers through this publication and presentation at scientific conferences and symposia.

### Grading level of evidence

The GRADE (Grading of Recommendations, Assessment, Development, and Evaluation) approach [56] will be adopted in weighing the quality of evidence as presented by included studies. The approach assesses quality of evidence against five criteria; 1) study limitations, 2) inconsistency of results, 3) indirectness of evidence, 4) imprecision, and 5) publication bias. Other factors that might increase quality of evidence such as large magnitude of effect, plausible confounding and dose-response gradient are also considered.

## Supporting information

S1 Fig PRISMA-P 2020 flow diagram. The PRISMA Flow diagram shows the flow of studies from retrieval from electronic databases and other sources to selection of studies for inclusion in the systematic review.
(TIF)

S1 Table PRISMA-P (Preferred Reporting Items for Systematic Review and Meta-Analysis Protocols) 2015 checklist: Recommended items to address in a systematic review protocol. (PDF)
