# Supplementary figures and images for: Praziquantel-related visual disorders among recipients in mass drug administration campaigns in schistosomiasis endemic settings: Systematic review and meta-analysis protocol
Source: PLoS One. 2024 May 17;19(5):e0300384. doi: 10.1371/journal.pone.0300384 (PMC11101040; doi:10.1371/journal.pone.0300384)

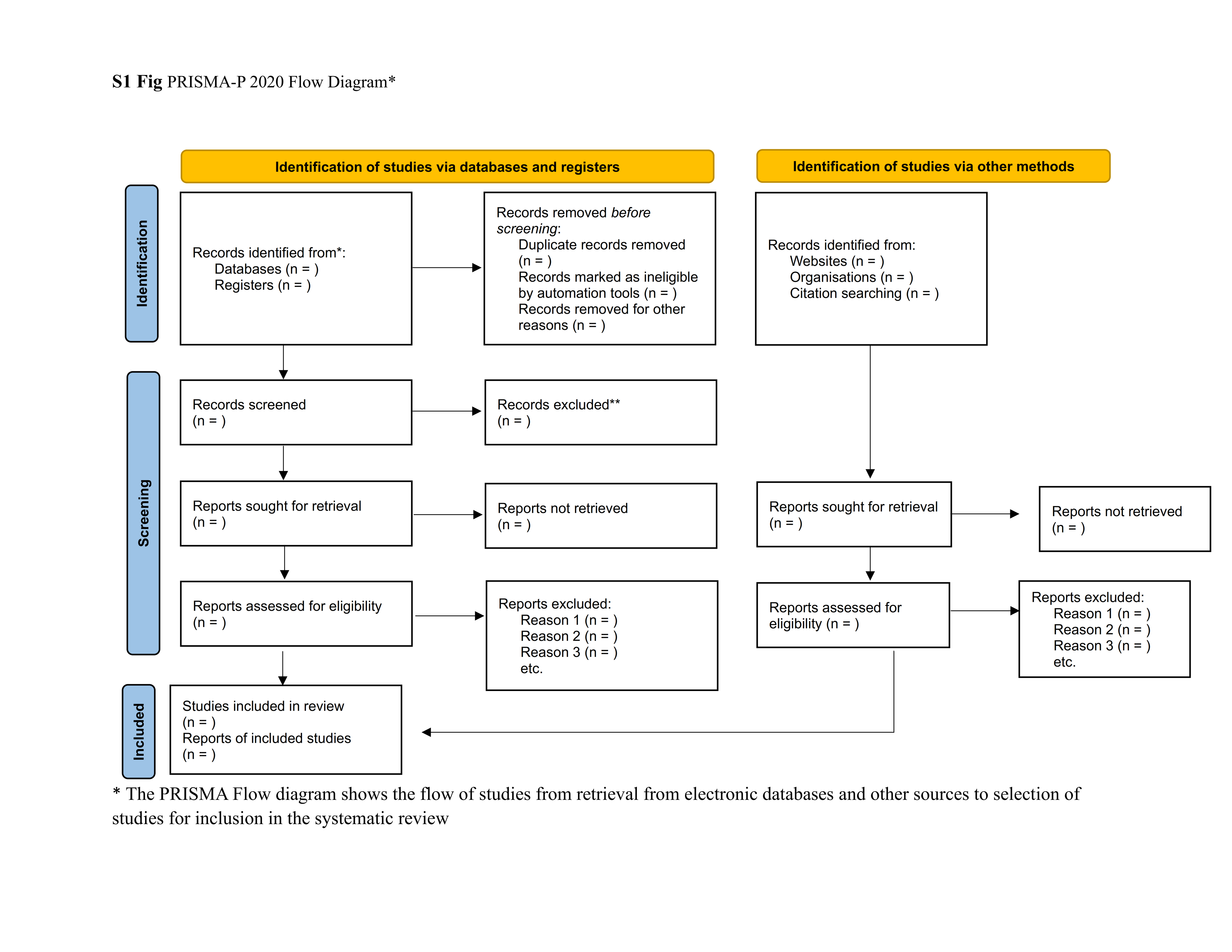

Supplement: S1 Fig — The PRISMA Flow diagram shows the flow of studies from retrieval from electronic databases and other sources to selection of studies for inclusion in the systematic review. (TIF) [file pone.0300384.s001.tif]
